# Study of Saponin Components after Biotransformation of *Dioscorea nipponica* by Endophytic Fungi C39

**DOI:** 10.1155/2022/2943177

**Published:** 2022-05-11

**Authors:** Nannan Huang, Dan Yu, Jinhai Huo, Junkai Wu, Yiyang Chen, Xiaowei Du, Xijun Wang

**Affiliations:** ^1^School of Pharmacy, Heilongjiang University of Chinese Medicine, Harbin, Heilongjiang 150040, China; ^2^Institute of Chinese Materia Medica, Heilongjiang Academy of Chinese Medicine Sciences, Harbin, Heilongjiang 150036, China

## Abstract

This study conducted the solid fermentation process of *Dioscorea nipponica* using endophytic fungi C39 to determine the changes in the diosgenin concentration. The results revealed that endophytic fungi C39 could effectively biotransform the saponin components in *D. nipponica*. Moreover, the maximum increase in the diosgenin concentration reached 62.67% in 15 days of solid fermentation. MTT assay results demonstrated that the inhibitory effects of the fermentation drugs on four types of cancer cells (liver cancer cells (HepG2), stomach cancer cells (BGC823), cervical cancer cells (HeLa), and lung cancer cells (A549)) were better than those of the crude drugs obtained from *D. nipponica*. The chemical composition of the samples obtained before and after the biotransformation of *D. nipponica* was analyzed by UPLC-Q-TOF-MS. A total of 32 compounds were identified, 21 of which have been reported in *Dioscorea* saponins and the ChemSpider database and 11 compounds were identified for the first time in *D. nipponica*. The biotransformation process was inferred based on the variation trend of saponins, which included transformation pathways pertaining to glycolytic metabolism, ring closure reaction, dehydrogenation, and carbonylation. The cumulative findings provide the basis for the rapid qualitative analysis of the saponin components of *D. nipponica* before and after biotransformation. The 11 metabolites obtained from biotransformation are potential active ingredients obtained from *D. nipponica*, which can be used to further identify pharmacodynamically active substances.

## 1. Introduction

The dried rhizome of *Dioscorea nipponica* is mainly used as a herb in China [1]; previous studies have reported that *D. nipponica* contains steroidal saponins, polysaccharides, starch, amino acids, and flavonoids, among which the main active ingredients are steroidal saponins, including dioscin, gracillin, trillin, protodioscin, and methyl protodioscin [2, 3]. They have various pharmacological properties, such as improving the cardiovascular system, regulating immune function, and exhibiting anti-tumor, anti-inflammatory, and analgesic activities [4–6]. They also have important developmental value. Clinically, they can be used for the treatment of arthritis, coronary heart disease, thrombosis, asthma, and tumors [7–9]. They are an important industrial raw material for the synthesis of steroidal hormone drugs and saponins that are used for cardiovascular diseases.

Biotransformation is an important method for the structural modification and transformation of active ingredients in traditional Chinese medicine [10]. Therefore, biotransformation technology can be used to effectively increase the content of saponins or produce new saponins in *D. nipponica*, which can reduce the shortage of *D. nipponica* and other *Dioscorea* herbal resources and improve the effective use of herbal resources. Biotransformation is practically significant for the industrial production, utilization, and conservation of medicinal resources. Endophytic fungi are a class of microorganisms that live inside healthy plant tissues and do not trigger the host plant to exhibit distinct symptoms of infection [11]. This characteristic makes them advantageous for their use in biotransformation. Endophytic fungi C39 isolated from *D. nipponica* was cocultured with ethanol extract of *D. nipponica* rhizome. The results showed a considerable increase in saponin content in the culture broth, and the molecular mechanism underlying the biotransformation of strain C39 was elucidated [12]. Moreover, the enzymatic catalysis of *Aspergillus oryzae* can be used to transform the crude drug obtained from *D. nipponica*, combining the enzyme production and activity in one simple and inexpensive method [13, 14]. The application of fermentation technology for the synthesis of drugs can modify the structure of the chemical components in Chinese medicine and increase or generate new medicinal effects to increase the range of its applications [15]. The total content of saponin from solid fermentation of *Dioscorea Aspergillus oryzae* was confirmed by *in vitro* cellular assay to decrease the survival rate of HepG2 cells in a dose-dependent manner and induce apoptosis in HepG2 cells, which indicated that the fermented product had an anti-tumor effect similar to that of *Dioscorea* [16]. The inhibitory effect of fermented ginseng products (FGP) from *Penicillium simplicissimum* GS33 on the growth of ovarian cancer cells ES-2 was determined experimentally. The results showed that FGP significantly inhibited the growth of ES-2 cells compared with ginseng extract and showed higher cytotoxicity, which significantly decreased cellular activity [17]. To conclude, biotransformation is important for increasing the content of chemical components, reducing the shortage of resources, and conserving the resources of wild medicinal plants. Therefore, biotransformation can become an effective strategy in the industrial production of potential drugs in the future.

Saponins are one of the important active ingredients of *D. nipponica*. However, because of their diverse composition, the extraction, separation, and structure identification methods cannot comprehensively reflect the saponins in *D. nipponica*. Therefore, a new method should be established for the rapid analysis of saponins in *D. nipponica* and to perform an in-depth analysis of its active ingredients. In the present study, we used ultrahigh performance liquid chromatography-quadrupole time-of-flight mass spectrometry (UPLC-Q-TOF-MS) [18, 19] for the rapid, effective, and qualitative analysis of saponins in the rhizomes and transformed products of *D. nipponica*. The results from the present study can be used as a reference for the material basis of the medicinal effects and quality control methods of *D. nipponica* and facilitate its further development and use as a medicinal resource.

## 2. Materials and Methods

### 2.1. Reagents

Methanol (analytical purity; Tianjin Comio Chemical Reagent Corporation, China), acetonitrile (chromatographic purity; Merck, Germany), formic acid (chromatographic purity; Fisher, USA), distilled water (Watson's Food & Beverage Corporation, China), fetal bovine serum (FBS; Zhejiang Tianhang Biotechnology Co. Ltd., China), penicillin-streptomycin solution (double antibiotics; Beyotime, China), pancreatic enzyme digest, 1640 cell culture medium, 3-(4, 5-dimethylthiazol-2-yl)-2,5-diphenyltetrazolium bromide (MTT), and dimethylsulfoxide (DMSO; Harbin Delishi Technology Co. Ltd, China). The herbal material of *D. nipponica* was purchased from Shiyitang Chinese Herbal Slices Co. Ltd. of Harbin Pharmaceutical Group and identified as the dried rhizome of *D. nipponica*; endophytic fungi C39; gastric cancer BGC823 cells; liver cancer HepG2 cells; lung cancer A549 cells; cervical cancer HeLa cells (Heilongjiang University of Chinese Medicine, Pharmacognosy Laboratory Storage); dioscin, protodioscin, pseudoprotodioscin, and diosgenin standards (111707–201402, 111937–201201, 111855–201402, 1539–200001; China Drug Biological Products Inspection and Certification Institute); and gracillin standard (15021011; Chengdu Master Biotechnology Co. Ltd.).

### 2.2. Experimental Methods

#### 2.2.1. Fermentation Process

Based on a past study report [20], the content of diosgenin increased considerably after the biotransformation of *D. nipponica* crude drug by the endophytic fungi C39. By measuring the content of diosgenin, the total spirostero saponins content is reflected. Briefly, 20.0 g of *D. nipponica* crude drug (sieved through 40 mesh) was precisely weighed and added to a 500 mL fermentation flask, to which 40 mL of distilled water was added and stirred well and infiltrate; then let it stand for wetting for 12 h, sterilized by autoclaving for 30 min, and then cooled to the room temperature to obtain the final sterilized drug. The resultant drug samples were inoculated with 5 mL of endophytic fungi C39 seed solution under sterile conditions and incubated under a constant temperature condition with humidity at 28°C for 5, 10, 15, and 20 days (*n* = 3), separately. The fermentation drug was transferred to evaporation dishes, baked at 50°C for 48 h until the sample dried and then set aside until further use.

#### 2.2.2. Saponin Extraction Process

The solid fermentation drug (equivalent to 2.0 g of crude drug) was precisely weighed and extracted by ultrasonication with 7 times the amount of 70% ethanol for 3 times, 2 h each time, followed by filtration, combined filtrate, centrifugation, and drying to recover the extract. The resulted extract was dissolved by ultrasonication in distilled water, and the solution was extracted thrice with an equal volume of petroleum ether and maintained in an aqueous layer extract. Then, the drug was extracted with an equal volume of water-saturated n-butanol, and the n-butanol layer was retained; the solution was extracted until it became colorless; and the n-butanol extracts were combined and then dried for recovery.

#### 2.2.3. Acid Hydrolysis Process

Hydrochloric acid (20 mL, 2.5 mol/L) was added to the obtained dry drug powder, refluxed in a boiling water bath for 3 h, and then cooled to room temperature. Then, add 20 mL of chloroform for extraction thrice; the chloroform layers were combined, followed by washing with distilled water until neutral and decolorization with an appropriate amount of activated carbon and filtration. The extract was recovered with chloroform to dryness, and the residue obtained was added to 10 mL methanol.

#### 2.2.4. Methodological Review

An appropriate amount of diosgenin reference substance was accurately weighed, and methanol was added to the same to prepare a reference substance solution of concentration 0.650 mg/mL and set aside. HPLC (Waters, USA) was performed under the following conditions: diamonsil C18 column (250 × 4.6 mm, 5 *μ*m), C18 guard column (Dikma), mobile phase 95% acetonitrile-5% water isocratic elution, column temperature 35°C, flow rate 1.0 mL/min, and detection wavelength 206 nm. Precision experiment: the above-mentioned diosgenin standard solution was absorbed, and five consecutive samples were injected, 10 *μ*L each time, and the peak area was measured.

Stability test: the solid 15-day fermentation drug was precisely weighed (equivalent to 2.0 g of crude drug), and the sample solution was prepared according to the operation methods described in Sections 2.2.2 and 2.2.3, and the drug was injected into the HPLC column at 0, 6, 12, 18, and 24 h, respectively, and 5 *μ*L of the injection material was used each time to determine the diosgenin content. Reproducibility experiment: 5 samples of 15-day fermentation drug were used to prepare the sample solution in accordance with the operation methods described in Sections 2.2.2 and 2.2.3, and 5 *μ*L of the reactants were used each time to determine the diosgenin content. Sample recovery experiment: the solid 15-day fermentation drug (equivalent to 1.0 g of crude drug) was precisely weighed and used to prepare the sample solution in accordance with the operation methods described in Sections 2.2.2 and 2.2.3; 7.765 mg of the diosgenin reference substance was used to prepare the test solution. The samples were injected separately (5 *μ*L) to determine the content of diosgenin, followed by the calculation of the sample recovery rate.

#### 2.2.5. Cell Culture and Proliferation Experiments

The fermentation process was followed as specified in Section 2.2.1, and the reaction was performed under constant temperature and humidity at 28°C over 15 days for the fermentation drug. The same operation was performed with the drug without any fungi, and the sterilized blank sample of the drug was used as the control drug.

The extraction method was performed as specified in Section 2.2.2. The substance was dissolved in distilled water, frozen in the refrigerator, and then freeze-dried to obtain the steroidal saponin extract. The saponin powder was prepared in the 1640 medium to a concentration of 250 mg/mL of masterbatch, 0.22 *μ*m filtered, debacterized as a stock solution, and diluted with the culture solution to the desired concentration before treatment. The same process was performed for the crude and control drugs.

Liver cancer HepG2 cells, gastric cancer BGC823 cells, cervical cancer HeLa cells, and lung cancer A549 cells were cultured in the 1640 medium supplemented with 10% fetal bovine serum and 1% double-antibody and cultured routinely in a cell culture incubator (Hong Kong Likang Co. Ltd.) incubated at 5% CO_2_ and 37°C. When the cells were in an exponential growth phase, 0.25% trypsin was used for digestion, and then a culture medium containing serum was used to terminate the effect of trypsin on the cells for subsequent experiments.

The cells at the exponential growth phase were diluted to 1 × 10^5^ cells/mL and added to a 96-well cell culture plate with 100 *μ*L/well, and 6 wells were replicated and incubated for 24 h. After the cells were plastered, the culture solution was discarded, and 100 *μ*L of drug-containing culture solution was added at the concentrations of 250, 125, 62.5, 31.25, 15.625, 7.8125, and 0 *μ*g/mL in 7 groups of concentrations. After 48 h of incubation, the supernatant was discarded; 100 *μ*L of the MTT solution (5 mg/mL) was added to each well; and the incubation was continued at 37°C for 4 h. MTT was discarded; 100 *μ*L of DMSO was added to each well; and the OD (optical density) value was measured at 491 nm on an enzyme label (ELISA, Thermo) after shaking for 15 min at room temperature. Cell inhibition rate (%) = [1 − A/*A*_0_] × 100% (*A*: mean value of each drug concentration group and *A*_0_: mean value of blank group).

#### 2.2.6. Preparation of UPLC-Q-TOF-MS Solution

The appropriate amount of dioscin, gracillin, protodioscin, pseudoprotodioscin, and diosgenin standard was weighed, and methanol was added to make the solution to approximately 50 *μ*g/mL as the standard solution. *D. nipponica* crude drug, 15-day fermentation drug, 20-day fermentation drug, control drug (the above samples were tested in 6 groups of parallel samples), and C39 fungi blank (3 groups of parallel samples measured) were used as the samples. Then, 2.0 g of *D. nipponica* crude drug (15-day fermentation drug, 20-day fermentation drug, control drug, and C39 fungi blank were all converted into 2.0 g of the crude drug), weighed precisely, and placed in a conical flask with a stopper, to which 25 mL of methanol was added, weighed, extracted by ultrasonication at room temperature (25 ± 1)°C for 60 min, and then cooled to make up for the weight loss. The solution was then stored at −4°C and centrifuged at 13,000 rpm for 10 min at 4°C before analyses, and the supernatant was extracted. The resultant supernatant was filtered through a 0.22 *μ*m microporous membrane, and the filtrate was transferred into a sample bottle and set aside until further use.

#### 2.2.7. Chromatographic and Mass Spectrometry Conditions

ACQUITY UPLC (Waters) and Waters ACQUITY UPLC BEH C18 column (2.1 × 100 mm, 1.7 *μ*m) and ACQUITY UPLC BEH C18 VanGuard Pre-Column (2.1 × 5 mm, 1.7 *μ*m) were used, with the column temperature set at 30°C, the mobile phase was composed of solution A (0.1% formic acid in water), solution B (0.1% formic acid in acetonitrile), and gradient elution (0–8 min, 95–5% B; 8–18 min, 70–30% B; 18–30 min, 40–60% B; 30–30.1 min, 0–100% B; and 30.1–35 min, 95–5% B); the flow rate was set to 0.3 mL/min, and the injection volume was set to 2 *μ*L.

We used the Triple-TOF 5600+ mass spectrometer (AB SCIEX, USA), the ionization mode was electrospray positive and negative ionization modes with ESI source, positive and negative ion source voltage of 5,500/−4,500 V, ion source temperature of 550°C, detection pressure (DP) of 80/−80 V, collision energy (CE) of 35/−35 eV, and collision energy spread (CES) of 15/−15 eV. The atomization gas was nitrogen with the 55 PSI for auxiliary Gas1, 55 PSI for auxiliary Gas2, and 35 PSI for cur gas.

#### 2.2.8. Data Analysis

Data acquisition software: Analyst TF 1.6 software (AB SCIEX); data processing software system: PeakView 2.0/MasterView 1.0 software (AB SCIEX); data analysis software: MarkerView (AB SCIEX), SPSS software 26.0 (IBM, USA). The primary mass spectrometry parent ion scan range was 80–1,600; IDA was set to the highest peak with a response value >100 cps for the secondary mass spectrometry scan; and the subsidiary ion scan range was 80–1,600 with DBS turned on.

## 3. Results

### 3.1. Quality Control and Changes in Diosgenin Content

**Figure 1:**
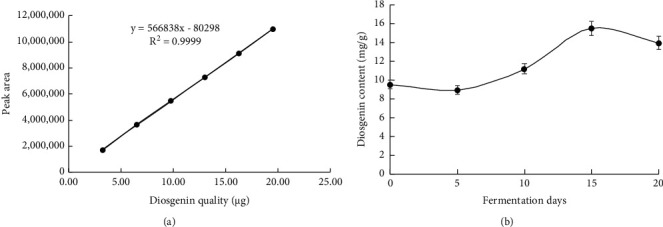
(a) Standard curve of diosgenin and (b) variation curve of diosgenin content on different fermentation days.

The chromatographic peak area *Y* and the standard quality *X* were used to plot a standard curve, and the linear regression equation obtained was as follows: *Y* = 566,838*X* − 80,298 (*R*^2^ = 0.9999), which indicated that diosgenin had a good linear relationship within the range of 3.25–19.50 *μ*g. The curve is shown in Figure 1(a). The relative standard deviation (RSD) of the content of diosgenin determined by the precision experiment was 0.87%, which confirmed the precision of the high-performance liquid chromatograph. The RSD calculated by the stability test was 0.38%, which indicated that the sample stabilized within 24 h. The RSD calculated by the reproducibility experiment was 0.07%, which indicated that the method had good reproducibility. The RSD calculated by the sample recovery experiment was 0.58%, and the average sample recovery rate of five determinations was 99.79%.

The fermentation was investigated on many days, namely 5, 10, 15, and 20 days of fermentation. The content of diosgenin in the fermentation samples increased from the 5^th^ day of fermentation and reached a peak at 15 days of fermentation. A downward trend was observed after 15 days of fermentation. The optimal number of days for solid fermentation was 15 days. The content of diosgenin was the maximum in the medicinal material fermented for 15 days, and the increase rate of diosgenin was 62.67%. The rate of change of diosgenin is shown in Figure 1(b).

### 3.2. Inhibitory Effects on Cancer Cells

**Figure 2:**
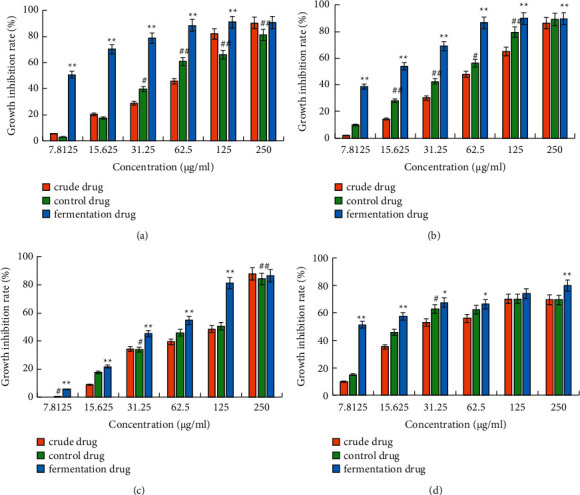
Inhibition of proliferation in different cancer cells by different concentrations of crude, control, and fermentation drugs: (a) effect on the viability of liver cancer HepG2 cells, (b) effect on the viability of gastric cancer BGC823 cells, (c) effect on the viability of cervical cancer HeLa cells, and (d) effect on the viability of lung cancer A549 cells. The values are presented as the mean ± SD (*n* = 6). ^*∗*^*p* < 0.05 and ^*∗∗*^*p* < 0.01 versus fermentation drug group and ^#^*p* < 0.05 and ^##^*p* < 0.01 versus control drug group.

In this study, the MTT assay was performed to detect the inhibitory effects of *D. nipponica* crude drug, sterilized control drug, and fermentation drug on the proliferation of liver cancer HepG2 cells, gastric cancer BGC823 cells, cervical cancer HeLa cells, and lung cancer A549 cells. The results showed that the inhibitory effects of the fermentation drug on all four types of cancer cell lines were more than those of the crude and control drug of *D. nipponica*. As shown in Figure 2(a), the inhibition rate of HepG2 cells in the 15.625 *μ*g/mL fermentation drug group was 70.320%, which was considerably higher than that of the crude drug group and the control drug group with the same concentration. A considerable difference was found when comparing the fermentation drug group with the crude drug group. Many cancer cell lines were treated with different concentrations of fermentation drug, and the results indicated that the fermentation drug inhibited the growth of various cancer cells in a dose-dependent manner within a certain concentration range. The results are shown in Table 1, and the inhibition rates are shown in Figure 2.

### 3.3. Determination of Differential Components

According to the liquid chromatography and mass spectrometry analyses, the sample was scanned using both positive and negative ion scan modes. The blank fungi solution was used as the blank standard, and ions with a corresponding intensity greater than 5 times the blank standard were extracted for target and nontarget screening.

Targeted screening: for dioscin compounds, the established primary mass spectrometry database of 109 saponins of *Dioscorea* species was used to set a mass error of <5 ppm, weight 30%, isotope difference <10%, weight 40%, Formula Finder score >70%, and weight 40% using the target screening function and characteristic subsidiary ion scan of the MasterView 1.0 software to screen component samples and fermentation samples [21].

Nontargeted screening: the collected data were imported to the MarkerView (AB SCIEX, USA) software, and Formula Finder was set to parent ion intensity of >1,000 counts, *S*:*N* > 10, and max element C_50_H_200_O_50_. All peaks in the data were extracted by the nontargeted screening function according to the set parameters. Then, the compounds with higher ion intensities were sorted by ion intensity, and the system displayed the XIC peak area variation of the ion within the component and fermentation samples.

**Figure 3:**
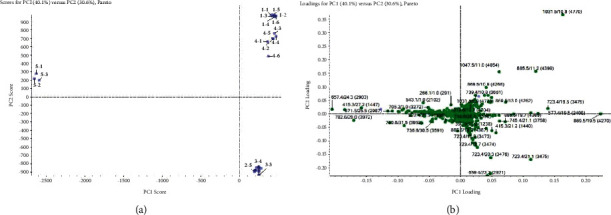
Score plot (a) and corresponding loadings plot (b) output from the principal component analysis. *Note.* 1: *Dioscorea nipponica* crude drug, 2: 15 d fermentation sample, 3: 20 d fermentation sample, 4: control drug, and 5: C39 fungi blank. Because some data were concentrated in the same area of 2–5, 3–3, and 3–4, the score plot data are not shown; therefore, the area of 2–5, 3–3, and 3–4 represents 2–1, 2–2, 2–3, 2–4, 2–5, 2–6, and 3–1, 3–2, 3–3, 3–4, 3–5, 3–6; the clustering situation is good.

In this study, different metabolites were selected on the basis of the generated raw data, data-preprocessing results, and principal component analysis results, and the different ions were identified on the basis of variable importance priority (VIP) values to filter out the different metabolites with a high contribution to the classification. In this experiment, the samples of *D. nipponica* crude drug, 15-day fermentation sample, 20-day fermentation sample, control drug, and fungi blank were tested and analyzed by PCA, and it was found that the difference between the *D. nipponica* crude drug and control drug was negligible, the changing trend in the 15- and 20-day fermentation samples was negligible, and the fungi blank sample had very little effect on sample detection. The differentiation effect was obvious for the *D. nipponica* crude drug and fermentation samples. The VIP value was used to identify the different metabolites, and the VIP value of >1.2 was screened to identify the different metabolites with a greater contribution. The principal component analysis is shown in Figure 3.

### 3.4. Structure Identification

**Figure 4:**
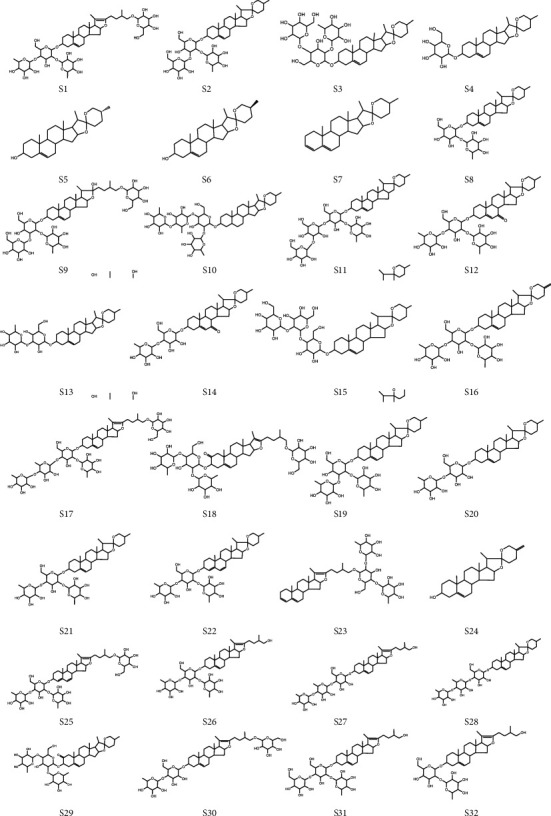
Structural formula of 32 saponins.

The differential ions were identified by principal component analysis using the MasterView function in the PeakView 2.0 (AB SCIEX) software to load the selected data. For the compounds that were compared with standards, the identification was performed by comparing the above information. However, for the compounds for which standards were not available, the possible chemical composition was inferred using Formula Finder for the excimer ion peaks given on the mass spectra. The structure of compounds was determined by searching and analyzing their mass spectral fragmentation characteristics, comparing the mass spectral fragmentation patterns of similar compounds, and combining the similarity of secondary spectra using ChemSpider [22]. Thus, we characterized 32 different molecules, including 21 compounds that have been reported in *Dioscorea* saponins compositions and the ChemSpider database and 11 compounds that have not been reported previously according to the specific molecular formula in *D. nipponica*, retention times, measured value, theoretical value, secondary fragment ions, and identification and variation trends as shown in Table 2 (note: ↑ or ↓ represents the up- or downregulation of the fermentation sample group compared with the crude drug sample group), and the structures of the compounds are shown in Figure 4.

### 3.5. Compound Mass Spectrometry Fragmentation Characterization

A total of 32 compounds were identified in this study, mainly including saponin components with similar cleavage pathways. Fragment characterization of previously reported and novel representative compounds was performed thereafter.

**Figure 5:**
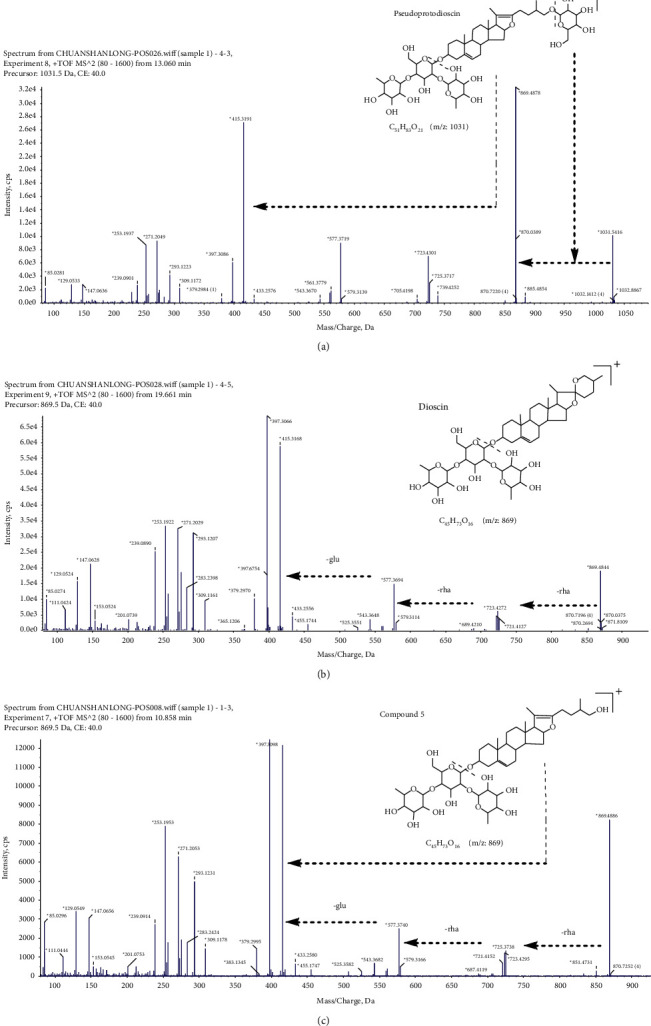
The MS/MS spectra of pseudoprotodioscin (a), dioscin (b), and compound 5 (c).

**Figure 6:**
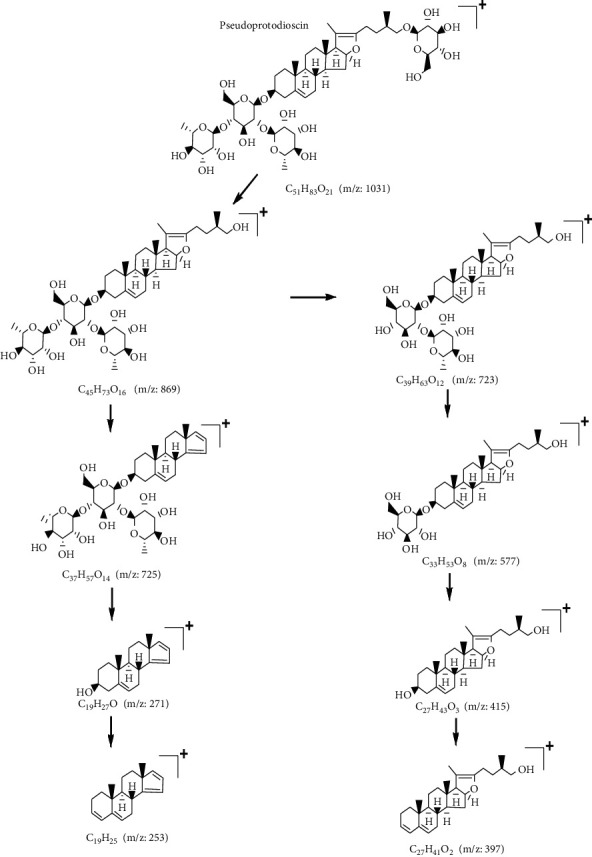
Fragmentation pathway of pseudoprotodioscin.

Pseudoprotodioscin (C_51_H_82_O_21_) in the ESI^+^ mode with an excimer ion m/z 1031 [M+H]^+^ first shed one molecule of glucose to form the fragment ion m/z 869 [M+H-C_6_H_10_O_5_]^+^. One of the next fragmentation pathways was as follows: after shedding the structure C_8_H_16_O_2_ to form the fragment ion m/z 725 [M+H-C_6_H_10_O_5_-C_8_H_16_O_2_]^+^, the three molecules of sugar were removed to form the fragment ion m/z 271 [M+H-C_6_H_10_O_5_-C_8_H_16_O_2_-C_6_H_10_O_4_-C_6_H_10_O_4_-C_6_H_10_O_5_]^+^, and finally, one molecule of H_2_O was removed to form the fragment ion m/z 253 [M+H-C_6_H_10_O_5_-C_8_H_16_O_2_-C_6_H_10_O_4_-C_6_H_10_O_4_-C_6_H_10_O_5_-H_2_O]^+^. The other fragmentation pathway was as follows: fragment ion m/z 869 shed rhamnose to form fragment ion m/z 723 [M+H-C_6_H_10_O_5_-C_6_H_10_O_4_]^+^; then, another rhamnose molecule was lost to form fragment ion m/z 577 [M+H-C_6_H_10_O_5_-C_6_H_10_O_4_-C_6_H_10_O_4_]^+^; then, glucose was removed to form the fragment ion m/z 415 [M+H-C_6_H_10_O_5_-C_6_H_10_O_4_-C_6_H_10_O_4_-C_6_H_10_O_5_]^+^, and finally, H_2_O was removed to form the fragment ion m/z 397 [M+H-C_6_H_10_O_5_-C_6_H_10_O_4_-C_6_H_10_O_4_-C_6_H_10_O_5_-H_2_O]^+^. Based on the fragmentation behavior of the standard and reference to literature reports [23, 24], ChemSpider website search for chemical structure features combined with the fragmentation characteristics of the compound was used to determine the compound as pseudoprotodioscin whose MS/MS spectra are shown in Figure 5(a), and fragmentation pathways are shown in Figure 6.

Dioscin (C_45_H_72_O_16_) in the ESI^+^ mode has an excimer ion of m/z 869 [M+H]^+^. Based on the fragmentation pattern of the standard and reference to literature reports [25, 26], ChemSpider website search for chemical structure characteristics combined with the fragmentation characteristics of the compound was used to determine the compound as dioscin. Its MS/MS spectra are shown in Figure 5(b).

Compound 5 (C_45_H_72_O_16_) had an excimer ion of m/z 869 [M+H]^+^ in the ESI^+^ mode, and one of the fragmentation pathways was the formation of the fragment ion m/z 725 [M+H-C_8_H_16_O_2_]^+^ after the removal of C_8_H_16_O_2_, the removal of three molecules of sugar to form the fragment ion m/z 271 [M+H-C_8_H_16_O_2_-C_6_H_10_O_4_-C_6_H_10_O_4_-C_6_H_10_O_5_]^+^, and finally, the removal of one molecule of H_2_O to form the fragment ion m/z 253 [M+H-C_8_H_16_O_2_-C_6_H_10_O_4_-C_6_H_10_O_4_-C_6_H_10_O_5_-H_2_O]^+^. The other fragmentation pathway was as follows: the fragment ion m/z 869 shed one molecule of rhamnose to form fragment ion m/z 723 [M+H-C_6_H_10_O_4_]^+^. The removal of another rhamnose molecule formed the fragment ion m/z 577 [M+H-C_6_H_10_O_4_-C_6_H_10_O_4_]^+^. Then, one molecule of glucose was removed to form the fragment ion m/z 415 [M+H-C_6_H_10_O_4_-C_6_H_10_O_4_-C_6_H_10_O_5_]^+^. Finally, one molecule of H_2_O was removed to form the fragment ion m/z 397 [M+H-C_6_H_10_O_4_-C_6_H_10_O_4_-C_6_H_10_O_5_-H_2_O]^+^. According to the chemical structure features retrieved from the ChemSpider website and the fragmentation features of the compound, its MS/MS spectra are shown in Figure 5(c).

### 3.6. Metabolic Pathways

**Figure 7:**
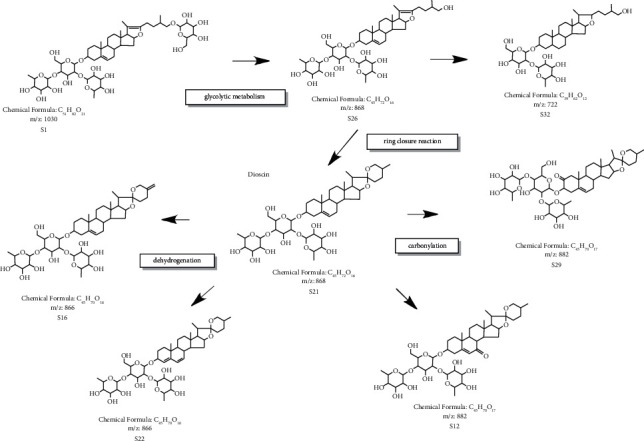
Various conversion processes.

Saponins with a molecular weight of more than 1,000 and 4 sugar molecules attached undergo different degrees of degradation. Verification of endophytic fungi can effectively break the glycosidic bonds of saponins to obtain saponins with a higher bioavailability and smaller molecular weights. In the case of pseudoprotodioscin, the change in molecular weight after sugar metabolism was 1030-868-722. The 26-position hydroxyl group in the S26 compound combines with the 22-position carbon, leading to a ring-closure reaction. The chemical structure confirmed that furostanol saponins are transformed into spirostero saponins, thus clarifying the cause of the increased diosgenin content. Centering on the S21 with a molecular weight of 868, the trend of dehydrogenation was evident in the transformation product with a molecular weight of 866, presumably with new structures, and relatively novel structures were also found in ChemSpider. Carbonylation was evident in the transformation product with a molecular weight of 882, such as the production of S12 and S29. Based on the above representative examples, it can be assumed that the conversion process included dehydrogenation and carbonylation processes, as shown in Figure 7.

## 4. Discussion

Although several studies have performed solid fermentation, our study was based on the increase in the amount of drug input and after methodological investigation, our method proved to be stable and feasible. Our experimental results showed that the samples that were fermented for 15 days had the largest increase in diosgenin content, and hence, the validation experiments were conducted using 15-day fermentation samples. Because of the sterilization operation step in the fermentation process, a sterilized blank sample was added as the control drug in the experiment for comparing and accurately determining the effect of fermentation and the crude drug on the cell inhibition rate. In the experiment performed for determining the variability of UPLC-Q-TOF-MS analysis samples, to accurately reflect the changes in the samples apart from adding the sterilization blank control drug, a group of samples with 20 days of fermentation was added to accurately identify the changes in the chemical composition of the samples after fermentation.

In this experiment, the research methods and ideas of plant metabolomics were applied to study the chemical composition changes in *D. nipponica* before and after biotransformation, and the UPLC-Q-TOF-MS analytical method was used to determine the chemical composition of *D. nipponica*. The raw data obtained were processed using the MarkerView software and analyzed by PCA. Using the literature data, standard controls, and secondary mass spectrometry fragmentation patterns, 32 saponins that contributed significantly to *D. nipponica* and its fermentation samples were screened and identified as chemical markers to distinguish *D. nipponica* before and after biotransformation. These saponin components were mainly dioscin, gracillin, prosapogenin A, trillin, and diosgenin, all of which had high activity [27–33], of which 21 compounds have been reported in *Dioscorea* saponins and the ChemSpider database [34–39], and the other 11 compounds were identified for the first time in *D. nipponica*. The structures were inferred by searching the ChemSpider website and analyzing the mass spectral fragmentation characteristics of the compounds, comparing the mass spectral fragmentation patterns of similar compounds, and combining them with the similarity of secondary spectra.

During biotransformation, the glycosidic bonds may be structurally broken by adding endophytic fungi, in which saponins with 4 sugars and molecular weights of more than 1,000 are degraded, such as pseudoprotodioscin, protogracillin, asperin, and zingiberensis saponin. Thus, we hypothesized that endophytic fungi can effectively degrade the glycosidic bonds and produce saponins with a higher bioavailability and smaller molecular weights. Dehydrogenation and carbonylation often introduced large amounts of organic reagents in chemical reactions, accompanied by the occurrence of side reactions, and the use of biotransformation allowed the structural modification of the target compound with minimal contamination. Structural analysis and the detection of content changes revealed that S12, S16, S22, and S29 variation trends were significantly higher, indicating significant dehydrogenation and carbonylation transformations. The changes in chemical composition by analysis can explain one of the reasons for the increase in diosgenin as the conversion of furostanoside to spirostanoside, which formed the basic skeleton of diosgenin and also contributed to the formation of some new saponin components. The saponin components in the converted fermentation samples showed effective inhibition rates against the 4 types of cancer cells and were more effective than the crude drug in a certain concentration range, indicating that the biotransformed saponins such as glycolysis, dehydrogenation, and carbonylation have better biological activity and provide an important basis for the discovery and application of new potent substances. In this study, we compared the changes in the chemical composition of *D. nipponica* before and after fermentation, analyzed and discussed its biotransformation process, which showed that the coincubation environment of *D. nipponica* and fungi C39 produced certain special enzymes [12], which in turn catalyzed the structural changes in chemical substances in the herbs, prompting the conversion of precursors in the original herbs to steroidal saponin components. The substantial increase in diosgenin supports this inference, in addition to the glycolysis, dehydrogenation, carbonylation, and cyclization reactions that occurred during the biotransformation process, which differentially increased or decreased the content of various chemical components, while new saponin components were generated. These may be responsible for significantly enhancing the anti-tumor effects of fermented samples of *D. nipponica* and also an important way to obtain potential medicinal active ingredients by biosynthetic transformation. Our results have practical implications for further understanding and revealing the biosynthetic process of saponins, as well as for the in-depth development and utilization of *D. nipponica* herbal resources. We mainly focused on the transformation of saponin components, and our study lacks a comprehensive analysis of the entire transformation process and the role of small molecule compounds in it, which needs to be further explored and discussed in future studies.

## 5. Conclusion

The endophytic fungi C39 can effectively biotransform the saponin components in *D. nipponica*. By examining the changes in the diosgenin content in the solid fermentation samples, we found that solid fermentation of 15 days had the largest increase in the diosgenin content (62.67%). The results of the MTT assay showed that the inhibitory effects of the fermentation drug on the four types of cancer cells (HepG2, BGC823, HeLa, and A549) were better than the crude and control drugs of *D. nipponica*, and the inhibition effect was significant in a certain concentration range. Chemical composition analysis of the samples before and after biotransformation of *D. nipponica* by UPLC-Q-TOF-MS identified 32 compounds, 21 of which have been reported in *Dioscorea* genus saponins and the ChemSpider database and 11 compounds were identified for the first time in *D. nipponica*. The biotransformation process was identified from the trend of saponin compounds, including sugar metabolism, ring closure, dehydrogenation, and carbonylation, providing a basis for studying the changes in the biotransformation of saponins components of *D. nipponica* by the endophytic fungi C39, as well as an important basis for the discovery and application of new pharmacophores.

## Figures and Tables

**Table 1:** The OD value of different drug concentrations.

		0 (*μ*g/mL)	7.8125 (*μ*g/mL)	15.625 (*μ*g/mL)	31.25 (*μ*g/mL)	62.5 (*μ*g/mL)	125 (*μ*g/mL)	250 (*μ*g/mL)
HepG2 cells	Crude drug	0.86 ± 0.03	0.81 ± 0.04	0.68 ± 0.12	0.61 ± 0.08	0.47 ± 0.03	0.15 ± 0.02	0.08 ± 0.01
	Control drug	0.85 ± 0.05	0.82 ± 0.07	0.70 ± 0.06	0.51 ± 0.07^#^	0.33 ± 0.06^##^	0.28 ± 0.06^##^	0.16 ± 0.05^##^
	Fermentation drug	0.89 ± 0.04	0.44 ± 0.09^*∗∗*^	0.26 ± 0.09^*∗∗*^	0.19 ± 0.05^*∗∗*^	0.10 ± 0.02^*∗∗*^	0.08 ± 0.01^*∗∗*^	0.08 ± 0.01
BGC823 cells	Crude drug	0.80 ± 0.05	0.79 ± 0.02	0.69 ± 0.04	0.56 ± 0.04	0.42 ± 0.05	0.28 ± 0.05	0.11 ± 0.02
	Control drug	0.85 ± 0.05	0.77 ± 0.04	0.61 ± 0.02^##^	0.49 ± 0.02^##^	0.37 ± 0.02^#^	0.17 ± 0.03^##^	0.09 ± 0.01
	Fermentation drug	0.83 ± 0.04	0.51 ± 0.02^*∗∗*^	0.38 ± 0.01^*∗∗*^	0.26 ± 0.02^*∗∗*^	0.11 ± 0.01^*∗∗*^	0.09 ± 0.01^*∗∗*^	0.09 ± 0.01^*∗∗*^
HeLa cells	Crude drug	0.93 ± 0.04	1.03 ± 0.03	0.84 ± 0.07	0.61 ± 0.03	0.56 ± 0.03	0.48 ± 0.05	0.11 ± 0.01
	Control drug	0.98 ± 0.03	0.98 ± 0.03^#^	0.81 ± 0.05	0.65 ± 0.03^#^	0.53 ± 0.02	0.49 ± 0.04	0.16 ± 0.02^##^
	Fermentation drug	0.93 ± 0.03	0.88 ± 0.06^*∗∗*^	0.72 ± 0.06^*∗∗*^	0.51 ± 0.03^*∗∗*^	0.42 ± 0.06^*∗∗*^	0.17 ± 0.05^*∗∗*^	0.12 ± 0.03
A549 cells	Crude drug	0.37 ± 0.02	0.33 ± 0.03	0.24 ± 0.02	0.17 ± 0.01	0.16 ± 0.02	0.11 ± 0.01	0.11 ± 0.01
	Control drug	0.40 ± 0.03	0.34 ± 0.03	0.22 ± 0.03	0.15 ± 0.02^#^	0.15 ± 0.02	0.12 ± 0.01	0.12 ± 0.01
	Fermentation drug	0.40 ± 0.03	0.20 ± 0.04^*∗∗*^	0.17 ± 0.02^*∗∗*^	0.13 ± 0.02^*∗*^	0.14 ± 0.01^*∗*^	0.11 ± 0.01	0.08 ± 0.01^*∗∗*^

Data are presented as mean ± SD (*n* = 6) ^*∗*^*p* < 0.05 and ^*∗∗*^*p* < 0.01 compared with fermentation drug group and ^#^*p* < 0.05 and ^##^*p* < 0.01 compared with the control drug group.

**Table 2:** Identification results and the trends of saponin components in the analyzed samples.

No.	*t*_R_/min	Measure value [M+H]^+^	Theoretical value [M+H]^+^	Error (×10^6^)	Molecular formula	Major secondary fragment ions select the ion [M + H]^+^	Identification	Variation trends
1	13.1	1,031.5416	1,031.5427	−1.1	C_51_H_82_O_21_	869.4878, 725.3717, 723.4301, 577.3719, 415.3191, 397.3086, 271.2049, 253.1937	Pseudoprotodioscin	↓
2	15.6	885.4802	885.4848	−5.2	C_45_H_72_O_17_	723.4287, 579.3115, 577.3684, 415.3201, 397.3096, 271.2057, 253.1952	Gracillin	↑
3	18.7	885.4804	885.4848	−5.0	C_45_H_72_O_17_	723.4282, 579.3111, 577.3707, 415.3195, 397.3090, 271.2055, 253.1948	Deltonin	↑
4	21.1	577.3718	577.3740	−3.8	C_33_H_52_O_8_	433.2588, 415.3195, 397.3104, 271.2066, 253.1961	Trillin	↑
5	26.5	415.3195	415.3212	−4.1	C_27_H_42_O_3_	397.3071, 271.2059, 253.1955	Diosgenin	↑
6	18.6	415.3217	415.3212	1.2	C_27_H_42_O_3_	397.3102, 271.2060, 253.1955, 147.1172	Yamogenin	↑
7	15.3	397.3108	397.3106	0.5	C_27_H_40_O_2_	379.3001, 253.1964, 147.1177	3, 5-deoxytigogenin	↑
8	15.3	723.4298	723.4319	−2.9	C_39_H_62_O_12_	579.3154, 415.3211, 397.3099, 271.2059, 253.1954	Prosapogenin A	↑
9	9.9	1,065.5384	1,065.5481	−9.1	C_51_H_84_O_23_	903.4887, 757.4301, 741.3665, 595.3782	Protogracillin	↓
10	12.1	1,015.5490	1,015.5477	1.3	C_51_H_82_O_20_	869.4898, 853.4949, 561.3757, 415.3205, 397.3098, 253.1945	Asperin	↓
11	11.8	1,045.5571	1,045.5583	−1.1	C_52_H_84_O_21_	883.5043, 737.4463, 575.3966, 429.3360, 253.1958	Zingiberensis saponin	↓
12	16.2	883.4714	883.4691	2.6	C_45_H_70_O_17_	737.4107, 591.3532, 429.3003, 411.2898, 285.1853	7-Oxodioscin	↑
13	15.9	723.4278	723.4319	−5.7	C_39_H_62_O_12_	579.3138, 577.3709, 415.3197, 397.3087, 271.2050, 253.1945	(3*β*, 25R)-spirost-5-en-3-yl3-O-(6-deoxy-*α*-L-mannopyranosyl)-*β*-D-glucopyranoside	↑
14	17.7	737.4100	737.4112	−1.6	C_39_H_60_O_13_	591.3521, 429.2995, 411.2891, 285.1849	*Dioscorea nipponica* saponin F	↑
15	11.1	901.4771	901.4797	−2.9	C_45_H_72_O_18_	739.4249, 577.3724, 415.3200, 397.3082, 271.2055, 253.1949	(3*β*, 25R)-spirost-5-en-3-yl*β*-D-glucopyranosyl-(1 ⟶ 2)-*β*-D-glucopyranosyl-(1 ⟶ 4)-*β*-D-galactopyranoside	↓
16	16.2	867.4728	867.4742	−1.6	C_45_H_70_O_16_	849.4625, 725.3728, 721.4165, 575.3552, 413.3044, 395.2934, 377.2830, 253.1939	(3*β*)-spirosta-5,25(27)-dien-3-yl6-deoxy-*α*-L-mannopyranosyl-(1 ⟶ 2)-[6-deoxy-*α*-L-mannopyranosyl-(1 ⟶ 4)]-*β*-D-glucopyranoside	↑
17	11.0	1,177.6012	1,177.6006	0.5	C_57_H_92_O_25_	1015.5440, 869.4860, 723.4282, 577.3717, 415.3187, 397.3083	Pseudoproto-Pb	↓
18	10.0	1,045.5188	1,045.5219	−3.0	C_51_H_80_O_22_	883.4659, 737.4080, 591.3501, 429.2990, 411.2893, 285.1849, 267.1747	(3*β*, 25S)-3-{[6-deoxy-*α*-L-mannopyranosyl-(1⟶2)-[6-deoxy-*α*-L-mannopyranosyl-(1⟶4)]-*β*-D-glucopyranosyl]oxy}-2-oxofurosta-5,20(22)-dien-26-yl *β*-D-glucopyranoside	↓
19	18.6	869.4820	869.4898	−9.0	C_45_H_72_O_16_	725.3685, 723.4259, 577.3688, 415.3179, 397.3076, 271.2044, 253.1937	Hypoglaucine A	↑
20	18.7	723.4292	723.4319	−3.7	C_39_H_62_O_12_	579.3149, 577.3717, 415.3199, 397.3092, 271.2057, 253.1953	Progenin II	↑
21	19.7	869.4844	869.4898	−6.2	C_45_H_72_O_16_	723.4272, 577.3694, 415.3168, 397.3066, 271.2029, 253.1922	Dioscin	—
22	17.5	867.4724	867.4742	−2.1	C_45_H_70_O_16_	721.4123, 575.3571, 413.3043, 395.2944	Compound 1	↑
23	18.7	851.4771	851.4793	−2.6	C_45_H_70_O_15_	705.4174, 559.3603, 397.3100, 253.1952	Compound 2	↑
24	16.4	413.3053	413.3055	−0.5	C_27_H_40_O_3_	395.2958, 377.2827, 269.1902, 251.1794	Compound 3	↑
25	10.6	1,029.5303	1,029.5270	3.2	C_51_H_80_O_21_	867.4760, 721.4175, 575.3578, 413.3059, 395.2955, 269.1904, 251.1799	Compound 4	↓
26	10.9	869.4886	869.4898	−1.4	C_45_H_72_O_16_	725.3738, 723.4295, 577.3740, 415.3204, 397.3098, 271.2053, 253.1953	Compound 5	↓
27	13.1	869.4858	869.4898	–4.6	C_45_H_72_O_16_	725.3724, 723.4290, 577.3706, 415.3184, 397.3078, 271.2041, 253.1935	Compound 6	↓
28	15.3	869.4853	869.4898	−5.2	C_45_H_72_O_16_	725.3719, 723.4287, 577.3713, 415.3189, 397.3088, 271.2056, 253.1950	Compound 7	↑
29	16.8	883.4653	883.4691	–4.3	C_45_H_70_O_17_	737.4080, 591.3508, 429.2987, 411.2887, 285.1848	Compound 8	↑
30	9.0	885.4835	885.4848	−1.5	C_45_H_72_O_17_	723.4312, 579.3161, 577.3732, 415.3212, 397.3102, 271.2063, 253.1956	Compound 9	↓
31	13.3	885.4816	885.4848	−3.6	C_45_H_72_O_17_	723.4275, 579.3124, 577.3703, 415.3176, 397.3073, 271.2032, 253.1922	Compound 10	↑, after↓
32	13.2	723.4304	723.4319	−2.1	C_39_H_62_O_12_	579.3149, 577.3727, 415.3196, 397.3098, 271.2058, 253.1951	Compound 11	↑, after↓

## Data Availability

The data used to support the findings of this study are included within the article.
